# Graphene–Bacteriophage Hybrid Nanomaterials for Specific and Rapid Electrochemical Detection of Pathogenic Bacteria

**DOI:** 10.3390/bios15070467

**Published:** 2025-07-19

**Authors:** José M. Campiña, António F. Silva, Carlos M. Pereira

**Affiliations:** Centro de Investigação em Química da Universidade do Porto—Institute of Molecular Sciences, Departamento de Química e Bioquímica, Faculdade de Ciências, Universidade do Porto, Rua do Campo Alegre s/n, 4169-007 Porto, Portugal; afssilva@fc.up.pt (A.F.S.); cmpereir@fc.up.pt (C.M.P.)

**Keywords:** graphene nanomaterials, bacteriophages, electrochemical biosensors, pathogen detection, bioreceptor immobilization, nanomaterial–bioreceptor interfaces

## Abstract

Efficient and rapid detection of bacterial pathogens is crucial for food safety and effective disease control. While conventional methods such as PCR and ELISA are accurate, they are time-consuming, costly, and often require specialized infrastructure. Recently, electrochemical biosensors integrating graphene nanomaterials with bacteriophages—termed graphages—have emerged as promising platforms for pathogen detection, offering fast, specific, and highly responsive detection. This review critically examines all electrochemical biosensors reported to date that utilize graphene–phage hybrids. Key aspects addressed include the types of graphene nanomaterials and bacteriophages used, immobilization strategies, electrochemical transduction mechanisms, and sensor metrics—such as detection limits, linear ranges, and ability to perform in real matrices. Particular attention is given to the role of phage orientation, surface functionalization, and the use of receptor binding proteins. Finally, current limitations and opportunities for future research are outlined, including prospects for genetic engineering and sensor miniaturization. This review serves as a comprehensive reference for researchers developing phage-based biosensors, especially those interested in integrating carbon nanomaterials for improved electroanalytical performance.

## 1. Introduction

Bacterial pathogens (BPs) can spread through direct contact between individuals, airborne droplets, ingestion of contaminated food or water, or via vectors like insects. Foodborne pathogens, such as *Listeria monocytogenes*, *Salmonella enterica*, or *Campylobacter jejuni*, cause serious gastrointestinal diseases [1,2,3]. In the European Union, *L. monocytogenes* caused 0.45 cases of listeriosis per 100,000 inhabitants in 2019, with a mortality rate of 17.6%, according to the European Food Safety Authority (EFSA) [2]. Globally, foodborne diarrheal disease agents, particularly non-typhoidal *S. enterica*, accounted for the majority of foodborne disease deaths, which totaled an estimated 230,000, in 2010. These agents also contributed to 33 million disability-adjusted life years [3]. This immense health burden translates into a significant economic impact, with global costs exceeding USD 110 billion annually [4], and it is driven primarily by healthcare expenses and productivity losses. Beyond foodborne diseases, crops are frequently devastated by pathogens such as *Ralstonia solanacearum*, *Pseudomonas syringae*, or *Xanthomonas* spp., resulting in significant food losses and insecurity [5]. In healthcare, coagulase-negative staphylococci cause infections in orthopedic implants, intravenous catheters, and prosthetic heart valves [6].

Furthermore, contamination of water resources with *Shigella*, *Campylobacter*, *Escherichia coli*, or *Vibrio cholerae* may trigger outbreaks of waterborne diseases with catastrophic effects, particularly in developing countries [7]. These examples highlight the urgent need for affordable, fast-responsive, sensitive, and selective analytical platforms capable of distinguishing between live and dead bacterial cells. The development of such devices is crucial to implement real-time on-site monitoring in food, medical, and water facilities, helping to prevent outbreaks, reduce food waste, and minimize antibiotic misuse [8]. Conventional microbiological analysis is highly accurate, but also laborious and time-consuming, requiring highly skilled personnel and often taking days to provide results. Modern immunological and nucleic acid amplification techniques, such as ELISA or PCR, have revolutionized disease diagnosis with faster results and lower detection limits. However, these methods rely on sophisticated equipment and face important challenges, such as the cross-reactivity of polyclonal antibodies or the inability of PCR to distinguish viable pathogens from dead cells [9,10].

In parallel, electrochemical biosensors (EBs) have emerged as promising platforms for the quantitative detection of bioanalytes, offering exceptional analytical performance [11], with minimal sample consumption in the microliter range [12], and seamless integration into portable devices [13]. These biosensors often benefit from the incorporation of nanomaterials (e.g., metals and carbon-based materials), which provide several advantages over their bulk counterparts. For instance, nanomaterials offer higher surface-to-volume ratios, enabling the immobilization of greater densities of bioreceptors, enhanced electrocatalytic properties, and reduced fabrication and operational costs. Additionally, quantum effects can lead to unique electronic, optical, and magnetic properties not observed in bulk materials [14]. Consequently, numerous electrochemical biosensor designs have been developed for detecting a wide range of bacteria, with a particular emphasis on nucleic acid- and antibody-based approaches [15,16,17,18,19,20]. For nucleic acid-based biosensors (also called genosensors), single-stranded DNA/RNA sequences, known as aptamers, specifically bind to their complementary strands in hybridization events occurring at the sensor’s surface, there by modulating its electrochemical response. This strategy is distinguished by its simplicity, versatility, rapid detection, and ability to discriminate specific targets.

Numerous aptamer-based EBs have been reported in the literature for detecting *E. coli*, *Pseudomonas aeruginosa*, and *V. cholerae*, among others [15,16,17,18,19,20]. These sensors have consistently demonstrated low limits of detection (LOD), ranging from 550 down to 1 CFU·mL^−1^, and high specificity toward their target analytes. However, while aptamers are generally regarded as potentially cost-effective, the production of custom aptamers is time-consuming and remains costly at the present time, particularly for niche or novel targets, limiting their commercial implementation [21,22]. Similarly, monoclonal antibodies face significant manufacturing complexities, including the need for genetically engineered cell lines, large-scale bioreactor culturing, multi-step purification, and stringent quality control [23,24]. These challenges currently hinder their large-scale production and contribute to high costs. As such, although numerous biosensors for pathogenic bacteria have been developed at the research level, their commercialization remains limited due to challenges in scalability, cost-effectiveness, and adaptability for complex samples. This gap highlights the need for innovative approaches that join high analytical performance to target specificity, low cost, and practical implementation.

**Figure 1:**
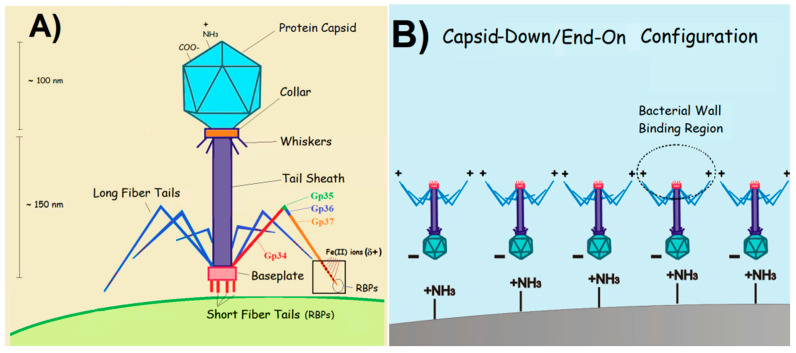
Artistic models: (**A**) the typical structure of a tailed bacteriophage, with receptor binding proteins (RBPs), located at the tip of the long fiber protein Gp37 (orange fragment); (**B**) a capsid-down (or end-on) orientation of tailed phages adsorbed onto a positively charged surface. Both illustrations are adapted with permission from P. M. V. Fernandes et al. [35]. Copyright 2023 MDPI.

Bacteriophages, or phages, are a distinctive class of viruses renowned for their precision in targeting and killing bacteria [25]. They can hone in on a narrow spectrum, ranging from multiple bacterial strains to specific serovars [26]. Selective recognition arises from the ability of phages to bind only to strain- or serovar-specific structures located on the bacterial membrane—such as lipopolysaccharides, teichoic acids, or outer membrane proteins—which is achieved via the molecular complementarities between these receptors and the binding domains of the phage [27]. These domains, located at the tail tip or lateral fibers (Figure 1A), contain receptor-binding proteins (RBPs), whose structural configuration determines host specificity. Recognition typically begins with interaction at a primary receptor, followed by irreversible binding to a secondary receptor that triggers tail sheath contraction and penetration of the bacterial envelope. By recognizing and binding to these structures, phages inject their genetic material into their hosts, effectively hijacking the molecular machinery of host cells [26,27,28]. For virulent phages, the takeover occurs within minutes, and the continuous assembly of new phages ends up breaking the host cell (lysis) and releasing the progeny [29]. Tailed phages account for about 96% of all known phages in nature [28], and their typical morphology is illustrated in Figure 1A. It consists of an icosahedral capsid that encases the genetic material, and a flexible tail structure responsible for host recognition and injection of DNA or RNA into the cells [28]. Over the past decade, researchers have integrated phages with electrochemical transducers to create phage-based EBs (PEBs), a novel class of biosensors with the potential to distinguish between viable and non-viable (dead) cells [30].

While PCR amplifies the DNA from both types of cells and ELISA detects antigens irrespective of viability, phages require specific receptors and active bacterial metabolism that are present only in live cells [28,29,30]. Such a unique feature, not achievable in geno- and immunosensors, is particularly crucial for accurately assessing contamination risks in food safety (as dead bacteria are not infective). PEBs also offer strong responses to specific target analytes at low cost, are easily implemented in portable platforms suitable for diverse settings [31,32,33,34,35,36,37,38], and have garnered extensive attention in the area of pathogen detection. As discussed above, nano-sized materials offer distinct advantages over macroscopic materials in biosensing applications. In particular, pristine graphene is known for its outstanding electrical and thermal conductivity, high optical transparency, mechanical strength and flexibility, and good compatibility with biological species [39,40,41]. These properties turn graphene nanomaterials (GNMs) into excellent candidates for biosensor performance and functional ranges. In recent decades, efforts have been devoted to harness the properties of GNMs into the realms of biosensors, batteries, mechanically reinforced materials, photovoltaic devices, and others [41,42,43,44,45,46,47].

High carrier concentrations in graphene facilitate efficient electrical communication between biological receptors—responsible for target-specific binding—and the electrode surface in graphene-based electrochemical biosensors (GEBs). This contributes to exceptional electrical transduction properties and strong responses to target analytes. For a comprehensive understanding and deeper insights into the diverse applications and performance metrics of GEBs, readers are encouraged to consult authoritative reviews in the field [41,44,45,46,47]. On the other hand, their large specific areas allow for the accommodation of higher loads of bioreceptors, leading to heightened sensitivities. The structural integrity and functional efficacy of biological entities, such as proteins, can be compromised when they establish strong interactions with supporting surfaces. For instance, proteins adsorbed onto certain solid surfaces may undergo conformational changes, leading to unfolding and loss of biological activity [48]. This phenomenon is influenced by the nature of the surface; highly polar interfaces tend to minimally perturb protein structure, whereas more apolar surfaces often significantly alter protein stability. However, the remarkable biocompatibility demonstrated by most graphenes is crucial for optimal EB performance [49].

Graphene and bacteriophages were first combined in 2010 by Cui et al. [50], marking the emergence of a novel class of hybrid nanomaterials. Despite their promising potential, it was not until the second half of the past decade that the first practical applications were reported [51]. The term graphages was coined in 2019 by P. Passaretti et al. [52]. A strong indication that research on graphages is still at a nascent stage, is the fact that Passaretti et al.’s study remains the only one to date that has systematically investigated the self-assembly and structural properties of graphene–phage hybrids. It demonstrates that graphage-based sponges exhibit a porous structure, ultra-low density, high surface area, and superior dispersion in aqueous environments, holding immense potential across different fields. Although further research is still needed to deepen our understanding of the fundamental properties of graphages, their ability to combine the exceptional properties of GNMs and phages opens new opportunities in different applications. Among these, potential uses include antimicrobial coatings. In such applications, graphages can leverage phages’ bactericidal properties to combat antibiotic-resistant pathogens [26,29,53].

Additionally, they may serve as functional materials for water purification membranes [54], exploiting their high porosity and bio-recognition capabilities to remove specific contaminants. These properties also make them attractive for drug delivery systems, offering targeted therapeutic delivery while benefiting from graphene’s biocompatibility, porosity, and tunable surface chemistry [55]. As discussed above, graphene is well-suited for use in biosensors [44,45,46,47]. However, when combined with phages or their receptor binding proteins (RBPs), this hybrid approach is expected to allow biosensors to maintain the low detection thresholds enabled by GNMs [44,45,46,47] while significantly enhancing target discrimination (thanks to the narrow range of most phages) [25,26]. Graphene’s high surface area and conductivity can be harnessed to immobilize phages in an orientation and density that maximize target capture and electronic readout. In this context, graphage-based EBs (GPEBs) have recently emerged [51,56,57,58,59,60,61,62] as a promising class of sensors with vast potential for quantifying pathogens across a wide range of sample types.

The results exhibited by PEBs fabricated with other phage–nanomaterial combinations [31,32,33,34,35,36,37,38] underscore the potential of GPEBs. The convergence of graphene’s advanced material properties with the biological targeting capabilities of phages is thus highlighted in the literature as a highly promising direction for next-generation biosensors [63,64]. However, despite such a disruptive potential, relatively few studies have investigated this area [51,56,57,58,59,60,61,62], which remains largely underexplored. This article provides a comprehensive overview of the advancements reported to date in the field of GPEBs. The discussion focuses on key aspects, including the prevalent types of GNMs and phages utilized, the conjugation methods employed, the orientation of graphene-attached phages (or their RBPs), the most frequent signal transduction schemes, and the electroanalytical performance of the reported devices. By addressing these topics, this review aims to capture the breadth of current knowledge on graphages for electrochemical detection of pathogenic bacteria. The survey also addresses the future perspectives and challenges of GPEBs.

## 2. Search Strategy

A structured literature search was conducted to identify all published studies on graphage-based electrochemical biosensors (GPEBs). Searches were performed in major academic databases (PubMed, Scopus, and Web of Science) using various keyword combinations of “electrochemical biosensor”, “graphene”, and “bacteriophages”. The inclusion criterion was application to bacterial pathogen detection, excluding studies solely focused on non-bacterial targets or alternative sensor platforms. To enhance the precision of the search strategy, AI-assisted tools (Scholar GPT) were employed to refine search queries and to help identify studies that use unconventional phrasing, emerging terminology, or atypical sensor descriptions. This step was particularly helpful for capturing entries that might have been missed by Boolean logic. To account for recent developments, updated searches were conducted at different points of writing. The last search was completed on 11 March 2025. The results indicated the existence of eight sensors reported so far [51,56,57,58,59,60,61,62], with no GPEB-related reports published prior to 2016. To the best of our knowledge, this review encompasses all reported GPEBs published to date.

## 3. Results

**Figure 2:**
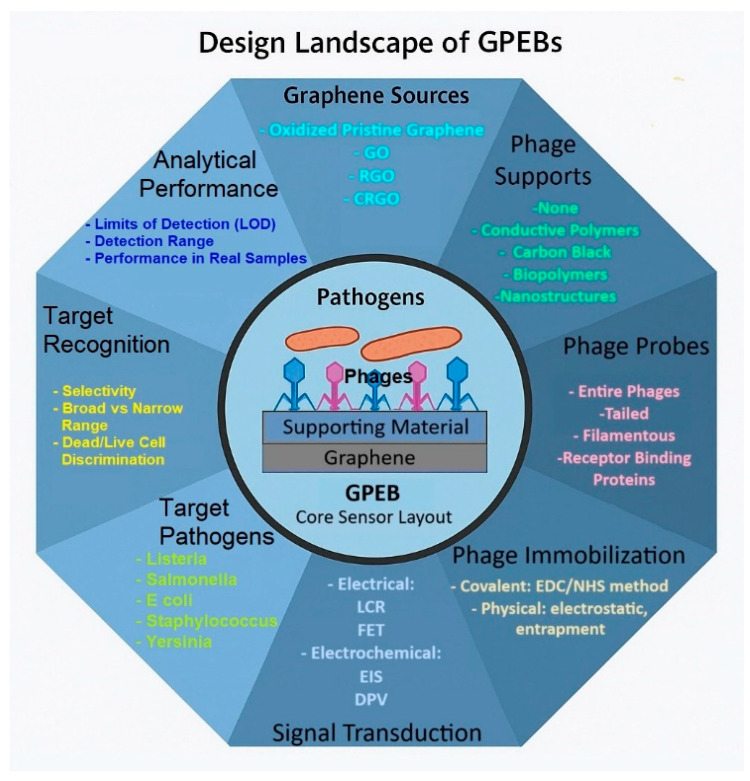
Conceptual framework illustrating the characteristics of GPEBs. At the center, a graphene surface functionalized with bacteriophages represents the biosensing interface. Radiating outward, eight interconnected domains summarize the key choices observed in the reviewed literature.

A critical step toward understanding the development of GPEBs is identifying the key design domains that define their architecture and performance. Figure 2 provides a conceptual synthesis of strategies and biosensor configurations reported in the eight experimental studies reviewed in this work. This schematic maps the actual combinations of materials, recognition elements, immobilization methods, and signal transduction approaches documented in the literature to date. Its core depicts a representative graphene-based interface decorated with bacteriophages (often using auxiliary materials such as conducting polymers or cellulose-based matrices). Surrounding the core, eight critical design domains were laid out: graphene sources, supporting materials, phage types, immobilization strategies, transduction methods, target pathogens, recognition specificity, and analytical performance. The Figure 2 helps clarify how different design decisions have shaped the capabilities and limitations of current GPEBs. These aspects are explored in detail in the following subsections.

### 3.1. Graphene Sources and Sensor Preparation

**Figure 3:**
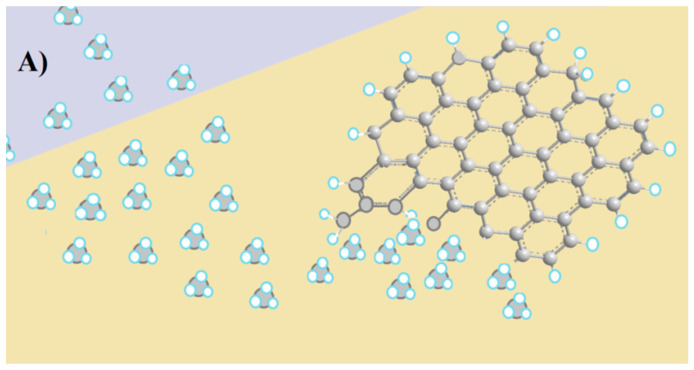

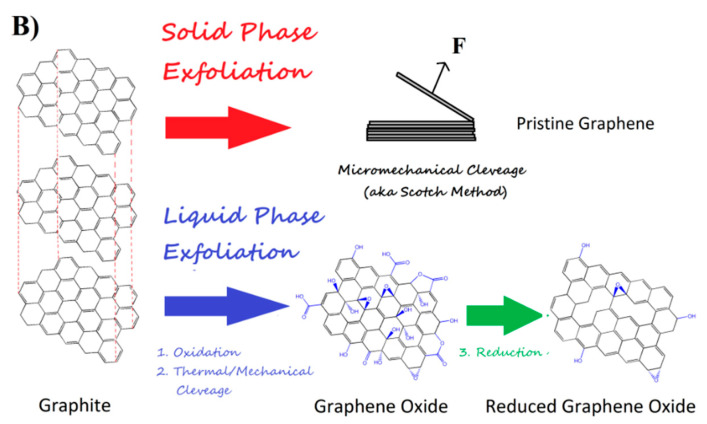
Comparison of the bottom-up (**A**) and top-down (**B**) approaches for synthesizing graphene nanomaterials: (**A**) Atom-by-atom growth of single-layer graphene sheets from organic molecules using vapor deposition processes. (**B**) Various methods employed for the exfoliation of graphite.

GNMs are generally produced through bottom-up or top-down strategies [65]. Bottom-up methods, such as chemical vapor deposition (Figure 3A), yield planar sp^2^-hybridized carbon structures—i.e., single-layer graphene—with exceptional electrical conductivity [66,67]. However, scalability and film transfer remain challenging [67,68]. In contrast, top-down approaches, like liquid-phase exfoliation (Figure 3B), provide scalable, cost-effective, and solution-processable routes [69]. These methods typically yield few-layer structures with wrinkled morphologies, higher defect density, lower electrical conductivity, and heteroatom doping (O, N, B, S, or P) [70,71]. Nonetheless, defects can enhance the electrocatalytic properties beneficial for specific applications [72,73].

In the context of GEBs, graphene oxide (GO) and reduced graphene oxide (rGO) have emerged as the most widely used GNMs produced through top-down approaches [41,44,45,46,47,72,73]. GO is heavily functionalized with oxygen-containing groups (see Figure 3B), which improve water solubility and provide abundant functional sites for covalent modification, but also render GO as electrically insulated [69,71,72]. Although GO cannot be fully reduced to restore the aromatic structure of pristine graphene [74], rGO offers a good balance between restored electrical conductivity and the electrocatalytic properties inherited from GO, making it a superior electrode material [75,76]. The performance of GEBs can be further enhanced by decorating GNMs with metal nanoparticles (NPs), metal–organic frameworks, or molecular imprinted polymers, increasing the surface area and density of electroactive sites [77,78]. These hybrid materials have been extensively applied in various platforms [79,80,81,82], highlighting their versatility for biosensing.

It is important to note that the overall response of GEBs is not only determined by the choice of graphene nano-material, as other factors—such as the electrode modification techniques (drop-casting, spin-coating, doctor blading, etc.) or the modification protocols (e.g., GNM-bioreceptor conjugate applied in a single step or through sequential layering)—also impact the structure, morphology, and integrity of the composite films [83,84]. The electrode preparation method can determine properties such as film uniformity, electroactive surface area, or phage stability, which in turn affect sensor performance [83]. Careful optimization of these aspects is also critical in the development of GPEBs. Despite the remarkable potential of graphage-based EBs in pathogen detection, only a few reported studies have explored their capabilities [51,56,57,58,59,60,61,62]. Table 1 summarizes the relevant aspects of these investigations, underscoring the predominant use of oxidized graphenes in these sensors.

**Figure 4:**
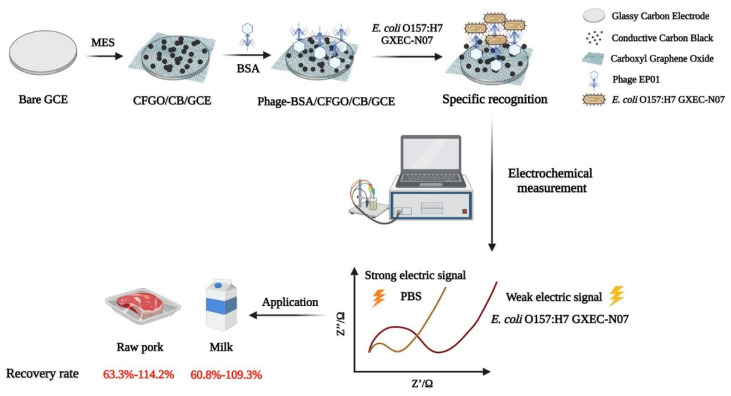
Workflow of a graphene–phage electrochemical biosensor developed by Zhou et al. [58] for the detection of *E. coli* O157:H7 GXEC-N07. The electrode was constructed by modifying a glassy carbon electrode (GCE) with carboxyl-rich graphene oxide (CRGO), conductive carbon black (CB), EP01 phages, and Bovine Serum Albumin (BSA). The figure illustrates the biosensor fabrication, bacterial recognition process, electrochemical signal transduction (via electrochemical impedance spectroscopy, EIS), and its analytical application in raw pork and milk matrices (including recovery rates). Reproduced with permission from Elsevier Science Ltd. Copyright 2021.

This preference can be attributed not only to the ease and cost-effectiveness of their synthesis, but also to their suitability for covalent attachment of phages and their binding proteins. For instance, in the seminal study of N. Bhardwaj et al. [51], commercial screen-printed electrodes (SPEs), made from pristine graphene inks, were functionalized by potentiostatic electrooxidation. Carboxyl (-COOH) groups were introduced and, later, chemically activated for covalent coupling of anti-*Staphylococcus arlettae* phages. GO has been often preferred since pre-oxidation steps are not required. To this end, Quiton et al. used commercial SPEs made from GO inks [56], and more recently, Y. Ding et al. [57] and Y. Zhou et al. [58] used chemically pretreated forms of GO with vast amounts of carboxyls. A detailed schematic of the fabrication and sensing workflow of the latter study is presented in Figure 4.

Although rGOs present significantly lower densities of O-groups, they have been effectively implemented in graphage-based biosensors. For instance, K. Nakama et al. [59] deposited rGO sheets—produced by thermal reduction of GO—onto interdigitated gold electrodes and covalently attached M13 phages to them. Q. Yang et al. [60] employed rGO/polyindole-5-carboxylic acid (PI-5-CA) composites on GCE, functionalized with anti-*Yersinia pseudotuberculosis* phages. Similarly, Keihan et al. embedded anti-*E. coli* phages in carbon paste electrodes (CPE) made from graphite, chemically reduced rGO, and paraffin oil [61]. More recently, Hussain et al. developed a biosensor by integrating rGO and anti-*S.* Typhimurium phages within a composite of bacterial cellulose (BC) and polypyrrole (PPy) [62]. This hybrid material offers high conductivity and a large surface area, enabling sensitive detection of the target pathogen.

These examples underscore the prominent role of oxidized graphene derivatives as phage supports in GPEBs, enabling versatile covalent attachment of biological recognition elements. Their surface defects and functional groups support non-covalent interactions, while non-functionalized sp^2^ planes facilitate π-π stacking. Notably, heteroatom-doped graphenes (e.g., N- or S-doped) and hybrid graphene–NP composites have not yet been explored in GPEBs. This is particularly surprising given their demonstrated advantages in other electrochemical sensors, such as enhanced surface area, tailored surface chemistry, and catalytic properties. Such materials could significantly enhance biosensor performance by enabling more efficient signal transduction, improved bioreceptor immobilization, and greater robustness in complex samples, representing a promising yet underexplored avenue for phage-based sensors.

### 3.2. Immobilization Methods

**Figure 5:**
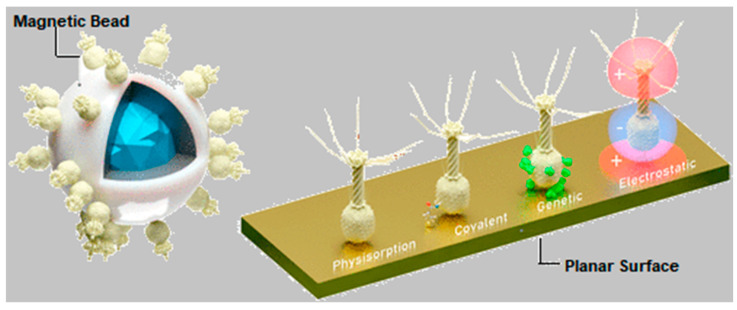
Artistic model illustrating the most common approaches for the immobilization of phages on surfaces. Adapted with permission from L. O’Connell et al. [86]. Copyright 2021 American Chemical Society.

**Figure 6:**
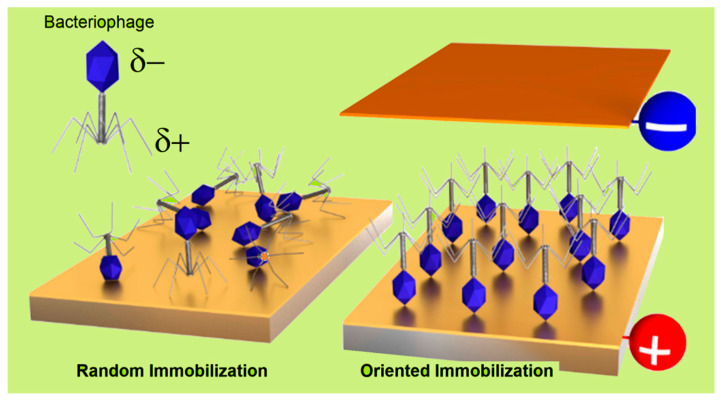
Schematic illustration depicting the electrical dipole nature of tailed bacteriophages and the deposition of their films by different methods. Phages immobilized by physisorption on a neutral surface or through unspecific covalent bonding (e.g., those targeting amino acid groups) typically result in a random orientation of phage constituents (**left** panel). Alternatively, the charge dipole that characterizes tailed phages allows for oriented immobilization in either tail-down or capsid-down (**right** panel) configurations. This can be achieved by applying an electric field with desired polarization [38] or by introducing charged groups on the substrate (as shown in Figure 1B). Adapted with permission from S.J., Machera et al. [92]. Copyright 2020, MDPI.

The application of phages in analytical, medical, or food safety settings often requires their attachment to a solid substrate. Selecting the appropriate immobilization method is vital for optimizing device performances. The most common strategies for immobilizing phages on surfaces are schematically illustrated in Figure 5 and are discussed below. In PEBs, maintaining the stability of the immobilized phages is essential to prevent detachment or structural unfolding, ensuring consistent and accurate sensor outputs over time. Surface-stabilized phages contribute to more reliable, long-term performance, while the proper alignment and accessibility of the receptor binding proteins (Figure 1B) are key for selectively capturing the target pathogens. Hence, controlling the orientation of asymmetrical species such as tailed phages—which constitute approximately 95% of all known phages [85]—is critical for maximizing binding efficiencies and minimizing non-specific interactions. The influence of phage dipolar properties on immobilization orientation is depicted in Figure 6. Additionally, the potential recovery and reusability of phages offer economic and environmental benefits, extending the lifespan of biosensors and reducing operational costs. Careful consideration of the physicochemical properties of phages is essential when designing immobilization strategies.

Most phages have a protein scaffold, known as the capsid, which encases their genetic material. Capsid proteins feature specific moieties, charge density, or polarities that facilitate chemical bonding and physical interactions with surfaces or linker molecules [87]. Key functional groups include primary amines (−NH_2_), carboxyls (−COOH), and sulfhydryl (−SH) groups in their constituent amino acids [88]. Therefore, by leveraging olarity (since tailed phages can be regarded as electrical dipoles) and local or overall substrate charge [89], effective immobilization strategies can be developed (see Figure 5 and Figure 6). Phages can also be genetically engineered using the phage display technique (PD) to express specific peptides or residues on their capsids [90]. PD expands the potential applications of phages by enabling tailored immobilization on various materials and the selective recognition of a greater variety of targets beyond the realm of bacteria [91]. Immobilization strategies can be generally categorized in three types: covalent, physical, and affinity binding.

#### 3.2.1. Covalent Bonding

Covalent methods represent one of the primary approaches for the immobilization of phages on solid surfaces. These usually involve intricate chemical reactions between specific functional groups on phages and substrates, resulting in robust and enduring attachments. Alternatively, the substrate can be modified with chemical linkers [93], such as silanes or thiols [94], facilitating its chemical coupling with functional groups on phage’s capsids [95,96,97]. Carbodiimide coupling using 1-ethyl-3-(3-dimethylaminopropyl) carbodiimide (EDC) and the N-hydroxysuccinimide method (NHS), a widely used strategy for anchoring other biological and supramolecular entities [98], has also been applied in phage attachment [92]. Carboxyl groups at the surface, either endogenous or introduced via linker modification, are activated by EDC to form intermediates that then react with NHS (which introduces succinimidyl groups). Primary amines on phage proteins attack these groups, leading to covalent amide bond-mediated immobilization [99,100]. Alternatively, carboxyl groups on capsid proteins can be activated to react with amino groups on the desired substrate.

Among the linkers used, the carboxyl- or amino-terminated alkylthiols that form self-assembled monolayers (SAMs) on gold surfaces are particularly popular as they yield highly stable attachments with strong resistance to environmental stress [92,101,102]. Despite the potential of covalent strategies, the abundance of reactive amino acids across the phage structure often results in random orientations (see Figure 6, left panel). Glutaraldehyde, a bifunctional cross-linker, has been employed as an alternative to EDC/NHS, forming stable amide bonds with primary amines on both phage and substrate under mild conditions [103,104,105]. These amines may be naturally present or introduced through linker molecules. For instance, He et al. used L-cysteine to anchor D29 phages onto gold electrodes [105], and Bhardwaj et al. functionalized metal–organic frameworks (MOFs) with phages following this approach [106,107]. In their pioneer GPEB design, the authors returned to the EDC/NHS strategy to immobilize anti-*S. arlettae* phages onto carboxyl-enriched graphene [51]. Carboxyl groups were activated by immersion in EDC and NHS, followed by incubation with an enriched phage solution (2 h, Table 1). The authors suggested that phages were predominantly oriented capsid-down to optimize RBP exposure (e.g., Figure 1B and Figure 6). However, no direct evidence supports this hypothesis and, in practice, random orientation remains highly likely due to the intrinsic characteristics of the EDC/NHS approach. Nonetheless, the method has become the preferred strategy for the fabrication of pathogen GPEBs. Quiton et al. also used EDC/NHS coupling to immobilize phages targeting *S. Enterica* Typhimurium on GO/carbon SPEs [56]. While they achieved good analytical performance, phage orientation was not investigated.

**Figure 7:**
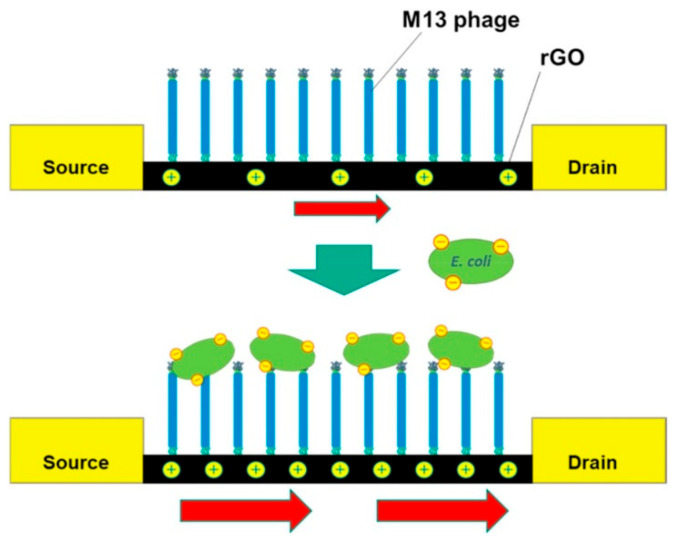
Design of the *graphage*-based field-effect transistor sensor reported by K. Nakama et al. for *E. coli* detection. The impact that pathogens captured by the rGO-M13 hybrid has on the electrical properties of the channel is also illustrated. Reprinted from [59]. Copyright 2021, Elsevier Science Ltd.

Nakama et al. [59] developed a graphage-based FET sensor using M13 phages immobilized on rGO via EDC/NHS chemistry (Figure 7). As in Quiton’s study, a longer incubation time was allowed to improve the density of immobilized phages. Although not experimentally verified, capsid-down orientation was inferred from the strong electroanalytical response. However, M13’s linear structure—with amino groups along the PVIII protein shell encasing their DNA—suggests random attachment is more likely. Zhou et al. introduced a one-pot EDC/NHS method to immobilize anti-*E. coli* EP01 phages onto carboxyl-rich GO (CRGO), along with carbon black, by co-incubating all components in MES buffer [59]. Components were co-incubated in MES buffer and drop-casted onto GCE (Figure 4). Ding et al. used a similar protocol to immobilize RBP41 proteins from T102 phages onto CRGO-coated GCEs [57]. In summary, while EDC/NHS chemistry ensures robust covalent immobilization, the abundance of reactive sites on phages often leads to uncontrolled orientation, potentially compromising sensor performance.

#### 3.2.2. Physisorption, Electrostatic Binding, and Affinity Binding

While covalent bonding ensures robust attachment, physical adsorption is often preferred for its simplicity [86], usually requiring only surface preparation and incubation with phage suspensions. However, these are based on weaker interactions—such as van der Waals and electrostatic forces, hydrophobic effects, or hydrogen bonding—which are highly sensitive to variations in pH, ionic strength, or temperature, and may cause partial phage desorption [95,108,109]. Among these approaches, electrostatic binding has gained particular interest [110,111,112,113,114,115,116,117]. To promote this, surfaces were modified to carry a net positive charge, typically using alkoxysilanes (e.g., APTES) or cationic polymers like poly-(L-lysine) (PLL), polyvinylamine (PVA), or polyethylenimine PEI [111,112,113,114,115,116,117], which maintain protonated amino groups (–NH_3_^+^) up to pH 9. Some of these linkers, such as—for instance—PEI, have also been used for the physical immobilization of pathogen-specific antibodies in immunosensors [36].

In tailed phages, the capsid surface typically carries a negative charge above pH 5 [118], favoring electrostatic interactions with positively charged substrates such as PEI-modified surfaces. This often results in capsid-down orientation, exposing the positively charged tails—rich in Fe-containing Gp37 proteins—toward the medium (Figure 1) [119]. While SAMs are commonly used for covalent coupling [92,101,102], recent findings suggest that amino-terminated alkylthiols can also promote electrostatic immobilization of anti-*L. monocytogenes* P100 phages in an RBP-exposing configuration [35]. This positions SAMs as suitable linkers for the electrostatic capture of phages, expanding their versatility to non-covalent methods (Figure 1B) and extending the range of feasible surfaces. Alternative physisorption strategies have been explored in the fabrication of other PEBs. Niyomdecha et al. physically entrapped Salmonella-specific M13 phages in polytyramine films [37]. Richter et al. implemented a hybrid approach combining alternating electric fields with covalent anchoring to immobilize T4 phages on gold surfaces [38].

**Figure 8:**
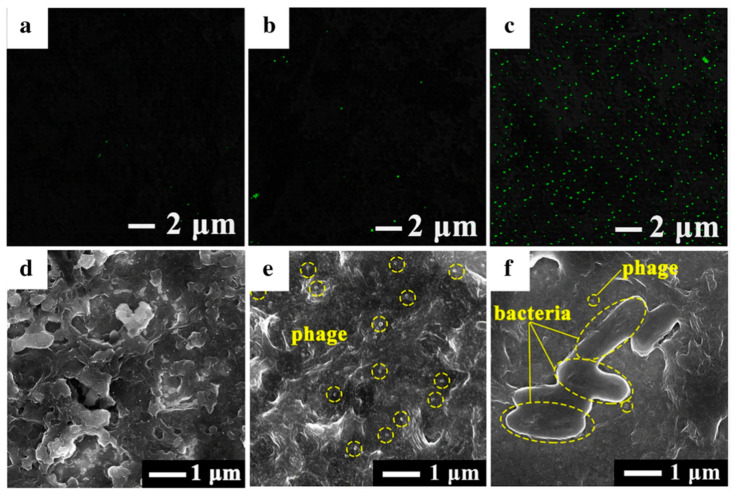
Confocal microscopy images depicting vB_YepM_ZN18 phages labeled with SYBR gold (a fluorescent nucleic acid stain) on various surfaces: (**a**) ITO/PI–5–CA, (**b**) ITO/PI–5–CA/rGO, and (**c**) ITO/PI–5–CA/rGO/AuNPs. FESEM images providing insights on the morphological changes of a GCE/PI-5-CA/rGO/AuNPs electrode (**d**), its transformation upon phage modification (**e**), and the subsequent capture of *Y. pseudotuberculosis* (**f**). Reprinted with permission from Q. Yang et al. [60]. Copyright 2021, Springer Nature.

In GPEBs, physisorption is less prominent than covalent bonding but still relevant. For instance, Yang et al. deposited vB_YepM_ZN18 phages and isolated them from hospital sewage onto a GCE modified with a PI-5-CA/rGO/AuNPs nanocomposite [60]. The sensor selectively detected the targeted *Y. pseudotuberculosis* species, confirming the viability of passive phage immobilization in such architectures. Fluorescence imaging of phage-modified GCE/PI-5-CA/rGO/AuNPs revealed significantly higher phage loading than on GCE/PI-5-CA/rGO (Figure 8b,c). Moreover, SEM images showed the formation of nanoparticle-rich porous surfaces (Figure 8d,e). These findings suggest the capture of phages via the weak Au–NH_2_ interactions established between phages and NPs. Although thisstudy did not explore this aspect, the widespread presence of –NH_2_ groups throughout the entire phage structure must likely render randomly orientated films. Another unresolved issue was whether PI-5-CA, if doped to carry a net positive charge, could also facilitate the capsid-down adsorption of phages, in addition to rGO sites, directly onto the polymer.

**Figure 9:**
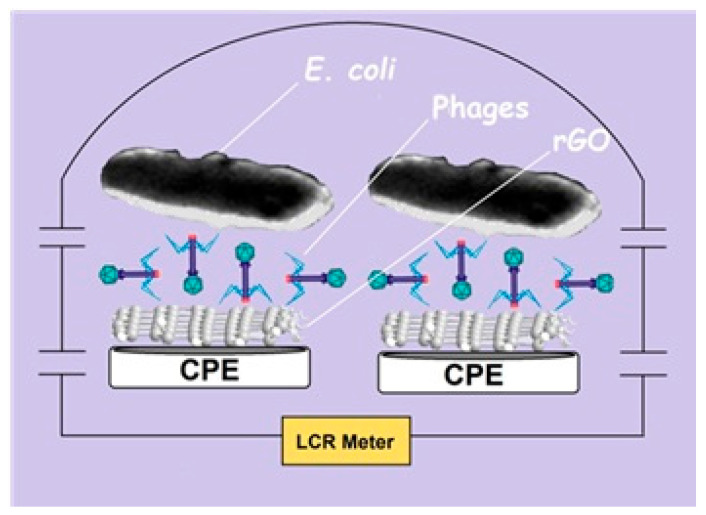
Simplified schematic of the experimental setup employed by A. H. Keihan et al. for the detection of *E. coli* XL1-blue [61]. The system comprised two planar carbon paste electrodes (CPEs), each modified with reduced graphene oxide (rGO) and bacteriophages and functioning as the plates of a capacitor. Prior to measurements, both CPEs were incubated in *E. coli* dispersions for predetermined durations. Capacitive measurements were conducted using a low-frequency LCR meter, with phosphate buffer acting as the dielectric medium.

This could improve the exposure of RBPs and enhance target recognition. The SEM images (Figure 8e) did not clarify phage configuration, and the relatively low phage density observed on GCE/PI-5-CA and GCE/PI-5-CA/rGO (Figure 8a,b) may reflect insufficient PI-5-CA doping. Further work is needed to investigate these effects. Hussain et al. immobilized anti-*S.* Typhimurium phages onto a rGO/PPy/BC-modified SPE using electrostatic interactions between the negatively charged phage capsid and the positively charged polypyrrole layer [62]. Although orientation was not addressed, the low LOD (1 CFU·mL^−1^) suggests high-density, capsid-down adsorption may have occurred—favoring efficient bacterial capture. A. H. Keihan et al. developed a capacitive sensor by mixing anti-*E. coli* phages with graphite powder, rGO, and paraffin oil to form a composite carbon paste electrode (CPE) [61]. Upon drying, the phages became physically entrapped within the graphitic structure. Due to the lack of orientation control, the authors suggested a random orientation of phages (Figure 9).

As further detailed in Section 3.4, this design could benefit from the inclusion of a positively charged polyelectrolyte to enhance phage orientation and accessibility. Another limitation is the restricted access of target bacteria to the entrapped phages, which are embedded primarily within the internal matrix of the electrode. This constraint may potentially reduce the electroactive area available for pathogens, impairing its ability to detect low concentrations and maintain linearity across multiple orders of magnitude. In addition to covalent and physical approaches, affinity binding via genetic engineering techniques has also been explored in phage immobilization. Introduced by G. P. Smith in 1985—the work that earned him the 2018 Nobel Prize in Chemistry—the PD method enables phages to be engineered to express short peptides on their capsid surface. These moieties act as targeting ligands for specific substrate residues [120]. This approach preserves the biological activity of phages and offers precise control over their orientation (both of which enhance biosensor performance). However, to our best knowledge, no GPEB has yet employed PD for immobilization, which highlights a promising avenue for future research. The negligible exploration of this approach likely reflects the high cost and technical demands of producing genetically modified phages, which involve complex protocols, regulatory hurdles, and specialized resources.

### 3.3. Signal Transduction and Detection Mechanisms

Electrochemical transduction remains a prominent strategy in biosensor design, although challenges persist in selectivity (particularly in complex biological matrices like blood or saliva), as well as in stability and reproducibility, due to the degradation of biorecognition elements and fabrication inconsistencies [121,122]. While optical sensors are advancing through femtosecond laser technology and miniaturized diffraction gratings [123,124], electrochemical platforms continue to dominate owing to their versatility and maturity [45,46,47,125]. The cost-efficiency, portability, fast response, and ability of EBs to operate label-free at trace-level concentrations make them ideal for on-site pathogen monitoring [126,127]. Often, biochemical recognition events modulate the electrochemical response in direct proportion to analyte concentration [128]. Amperometric and voltametric methods track variations in electrical current, while potentiometric and conductometric techniques measure changes in the electrode potential and conductivity, respectively [129]. Capacitive methods also detect target binding through shifts in the dielectric properties of the surface [128].

Electrochemical impedance spectroscopy (EIS) enables detailed characterization of interfacial phenomena by modeling electrode responses using simplified equivalent electrical circuits [129]. Of particular relevance is the charge transfer resistance (*R_CT_*), which reflects the kinetics of electron transfer (ET) across the interface [130]. This parameter can be modulated by factors such as electrode material, surface structure and morphology, and the nature of the electroactive species. Many biosensing strategies are based on the binding of non-conductive analytes to the surface, producing an increase in *R_CT_* that indicates hindered ET kinetics. FETs offer an alternative transduction mechanism, where shifts in the threshold voltage and drain current of the transistor are used to monitor probe–target binding interactions [41]. Hence, selecting the appropriate transduction strategy is crucial for the efficient conversion of bio-events into measurable electrical signals. It is essential to align the transduction mechanism and materials of choice with the specific application and targeted analyte [131].

Among the outlined possibilities, EIS has been the most popular choice in GPEBs to date (Table 1). This preference stems from its remarkable responsiveness to surface modifications and its rapid analysis (typically 10–15 min) [130,132]. The studies by Bhardwaj [51] and Quiton [56] clearly illustrate the effectiveness of EIS, showing strong correlations between pathogen concentration and electrode impedance. In these systems, the accumulation of large-sized bacteria captured by surface-immobilized phages physically blocks the surface, hindering the transport of redox species to the surface and disrupting the ET processes. This phenomenology results in a measurable increase in *R_CT_*, which is central to the detection mechanism of most impedimetric GPEBs. Higher pathogen titers in the incubating medium lead to greater surface coverage, amplifying impedance changes. By monitoring variations in *R_CT_*, the target can be quantitatively detected after appropriate calibration.

**Figure 10:**
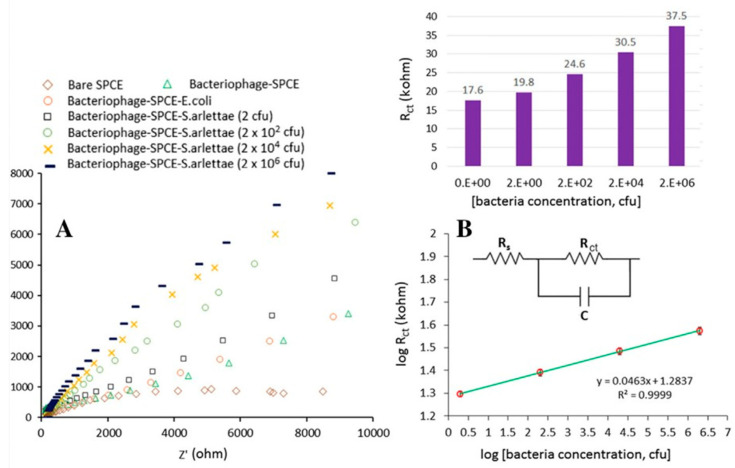
(**A**) Nyquist diagrams recorded using graphene-based screen-printed electrodes in the presence of 5 mM [Fe(CN_6_)]^+3/+2^ + 0.1 M PBS: unmodified electrode (wine diamonds), phage-coated electrode (green triangles), and electrodes exposed to *S. arlettae* at concentrations ranging from 2·10^2^ to 2·10^8^ CFU·mL^−1^ (red and green circles, black squares, yellow crosses, and black underscores). (**B**) Top: Charge transfer resistance (*R_CT_*) values derived from the data in (**A**). Bottom: Linear regression analysis of the top dataset in logarithmic format. Reprinted with permission from N.Bhardwaj et al. [51]. Copyright 2016, Elsevier Science Ltd.

**Figure 11:**
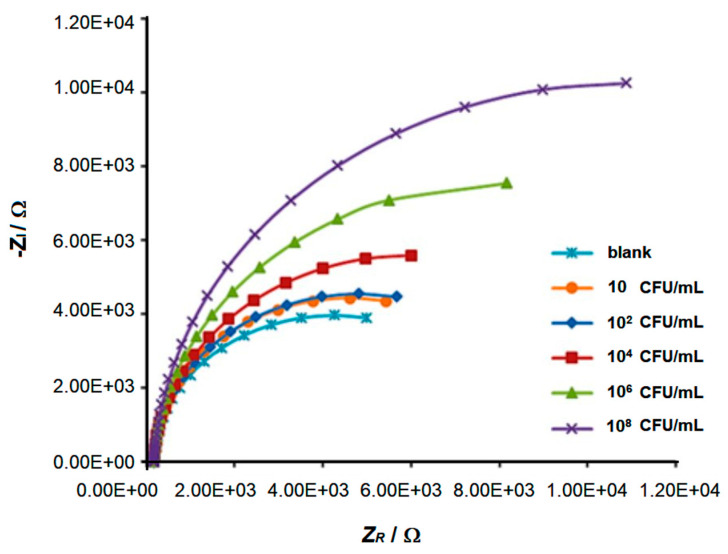
Nyquist diagrams recorded in 5 mM [Fe(CN_6_)]^+3/+2^ + 0.1 M PBS using GO/phage-modified screen-printed electrodes previously incubated in the following *S. enterica* solutions: non-incubated (cyan squares), 10 (orange circles), 10^2^ (blue diamonds), 10^4^ (wine rectangles), 10^6^ (green triangles), and 10^8^ CFU·mL^−1^ (violet crosses). Adapted from Quiton et al. [56] (CC BY license). International Frequency Sensor Association (IFSA) Publishing, S.L., 2018 (available at https://sensorsportal.com/HTML/ST_JOURNAL/vol_28.html; accessed on 15 June 2024).

In Bhardwaj’s study [51], graphage-modified electrodes were incubated with 10 µL of *S. arlettae* solutions ranging from 2·10^8^ to 2·10^2^ CFU·mL^−1^. The corresponding Nyquist plots (*Z_R_* vs. *–Z_I_*, Figure 10A) clearly reflect the magnitude of the bacterial concentration, with *log R_CT_* values showing a strong linear correlation with *log* [*S. arlettae*] (Figure 10B). A low LOD of 200 CFU·mL^−1^ was achieved, and the biosensor successfully detected *Salmonella* in spiked water and apple juice samples. Similarly, Quiton et al. used EIS to quantify *S.* Typhimurium [56], with Nyquist plots displaying a continuous increase in semicircle diameter (Figure 11). A strong linear correlation between *R_CT_* and bacterial titer was observed, and the sensor achieved a LOD 15 times lower than that reported by Bhardwaj et al. (Table 1). As shown in Figure 4, the biosensor developed by Zhou et al. [58] employed a layered architecture composed of carboxyl-rich graphene oxide (CRGO) and carbon black, forming a conductive and biorecognition–active matrix that supports EP01 phages.

This configuration amplifies impedance responses during bacterial recognition—a key factor aspect underpinning the sensor’s high responsiveness. Collectively, these findings underscore the effectiveness of impedimetric transduction linked to the phage-pathogen phenomenology described above. However, biosensor performance also depends on other factors, including the phage load, orientation, and activity; the specific GNM used; the conductivity of the nanocomposite; the electrode architecture; and the incubation time with the pathogen. For instance, in *E. coli* detection, reported LODs vary widely. Nakama et al.’s chemiresistor biosensor achieved a LOD of 45 CFU·mL^−1^ [59], while both Keihan et al.’s capacitive sensor [62] and Zhou et al.’s impedimetric device [58] reached significantly lower values, i.e., down to 12 CFU·mL^−1^. These differences reflect the complex interplay between the distinct sensing principles and design features determining the unique performance of each device.

**Figure 12:**
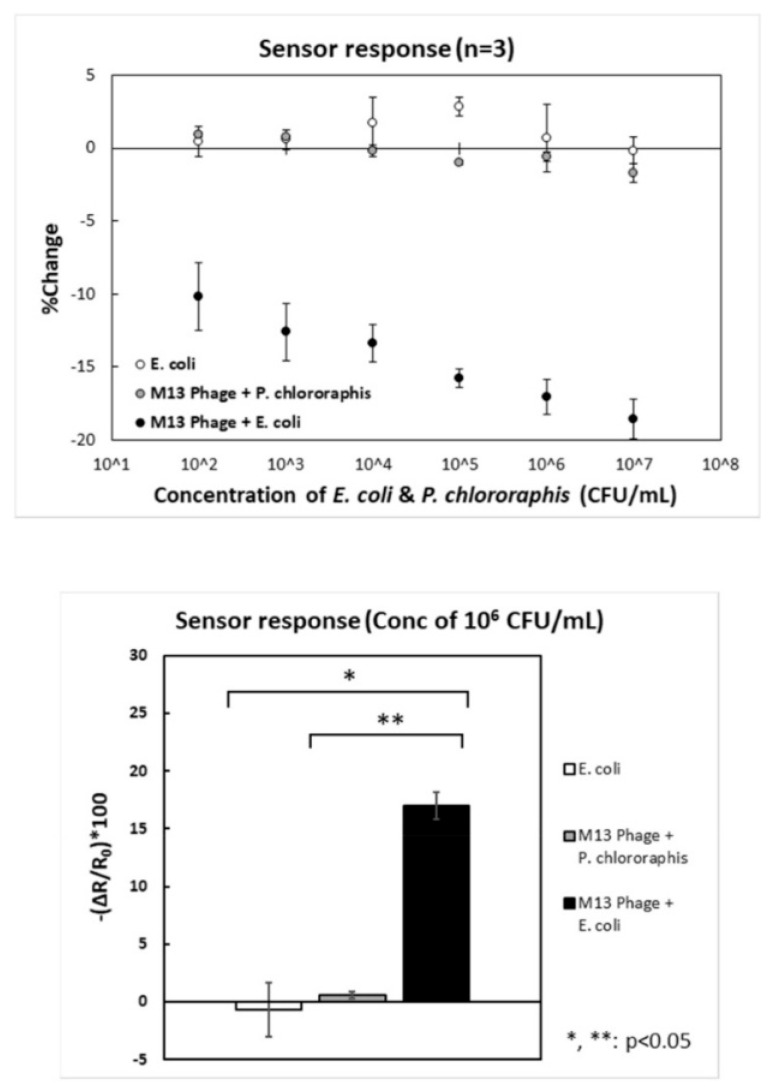
Resistance change % [(R − R_0_/R_0_) × 100] measured against the concentration of *E. coli* (host bacteria) and *P. chlororaphis* (control). The curves registered in the presence of *E. Coli* were taken with a GFET containing M13-modified (full black circles) and bare rGO channels (empty circles). R_0_ is the resistance measured after incubation with phosphate buffer (blank), and R is the value measured after incubation with the given bacterium (*E. coli or P. chlororaphis*). The control curve is shown in gray circles. The results obtained for a bacterial concentration of 10^6^ CFU·mL^−1^ are better compared in the bottom chart (same color key). Reprinted with permission from K. Nakama et al. [59]. Copyright 2021, Elsevier Science Ltd.

Further elaborating on sensing mechanisms, Nakama’s FET operates slightly differently [59]. First, channel-immobilized phages capture the target bacteria during incubation. Then, bacterial capture induces measurable changes in the channel’s resistance. The strong correlation between the incubation titer and resistance changes is shown in Figure 12. This evidence confirms the efficient conversion of bacterial capture events into proportional electrical signals. Keihan’s GPEB employed capacitive measurements, with two rGO/phage-modified CPEs positioned at a fixed distance in a buffer solution (the dielectric medium) [61]. Before *E. coli* capture, phages impart a net surface charge, resulting in measurable baseline capacitances at each interface. Upon pathogen binding, the charge becomes partially neutralized, leading to a decrease in total capacitance (ΔC). The sensor’s response showed a strong correlation with bacterial titer (R^2^ = 0.979), demonstrating that the amount of captured bacteria can be reliably translated into proportional capacitive signals.

However, the most sensitive GPEBs discussed in this review employed differential pulse voltammetry (DPV) [57,60,62], a widely employed technique in biosensing. As with impedance-based sensors, the detection principle relies on changes induced at the electrode surface upon target recognition. In Q. Yang et al.’s study [60], increasing the surface coverage of *Y. pseudotuberculosis* progressively reduced the current measured at the GCE/PI-5-CA/rGO/AuNPs/phage bioelectrode. In the absence of bacteria, cyclic voltammograms (CVs) and DPVs exhibited well-defined redox peaks, which were attributed to the activity of PI-5-CA. However, following incubation with the target, these signals were partially suppressed, indicating that pathogen binding interfered with ET processes. A similar current inhibition was observed by Hussain et al. [62], who used DPV to detect *S. Typhimurium* with a phage-functionalized rGO/PPy/BC composite. All three studies reported strong correlations between changes in the peak current (*Δi_P_*) and bacterial titer, achieving LODs below 5 CFU·mL^−1^ (Table 2).

### 3.4. Electroanalytical Performance

For effective monitoring of food and water safety, viable GPEBs must match or exceed the analytical performance of conventional methods. Key indicators include sensitivity, specificity, reliability, response time, and detection range [138]. Broad linear ranges and low detection limits are essential to quantify pathogens across relevant concentrations. Specificity is critical to avoid false positives and overestimation of contamination levels. Another key feature is the ability to discriminate viable from non-viable pathogens, as only live bacteria pose a health risk [139]. Effective operation in complex matrices is also crucial for real-world use [140]. Table 2 summarizes the analytical performance of the reviewed GPEBs along with other methods, such as cell culture, PCR, ELISA, and immunosensors [133,134,135,136,137].

GPEBs have rapidly approached the sensitivity of conventional culture methods, with minimum LODs ranging from 1 to 7 CFU·mL^−1^ [51,56,57,58,59,60,61,62]. Although their usability seems limited to 2–6 orders of magnitude, they offer a major advantage in analysis time, delivering results in about 30 min—versus the 2–4 days required by slow-growth-limited cell culture [137]. Only two studies have confirmed the ability to distinguish viable from dead bacteria [60,62], but future sensors are expected to incorporate this feature as phages cannot infect non-viable cells [139]. In contrast, conventional methods like PCR and flow cytometry (FC) lack this capacity [141]. For example, in H. Mohr et al.’s work [136], FC enabled simultaneous detection of up to 10 different pathogens but showed a high LOD of 10^5^ CFU·mL^−1^ and similar response times to culturing.

In addition, FC requires costly instrumentation and skilled operators [142]. While ELISA and PCR reduce analysis times to a few hours [134,135], graphage biosensors deliver reliable results in just 30 min, with comparable detection ranges and improved trace-level detection. Illustrating this view, Ding’s biosensor [57] showed a lower LOD for *S. enterica* Typhimurium than that reported by Wang et al. using quantitative PCR (qPCR) [134]. While most of the reviewed sensors took around 30–35 min to complete the analysis, Bhardwaj et al.’s sensor [51] detected *S. arlettae* in just 2 min. This result seems to be determined by the duration of the pathogen incubation step, i.e., 2 min vs. the 30 min allowed in most studies, and it also illustrates that analysis times are clearly influenced by this parameter.

The sensor achieved a detection limit of 200 CFU·mL^−1^, the highest among the reviewed studies, though it could likely be potentially reduced by extending the incubation time. Another noteworthy exception is Keihan et al.’s capacitive biosensor [61], which delivered results in just 5 s by eliminating incubation altogether. Its rapid response was attributed to both the absence of incubation steps and the intrinsic speed of LCR measurements. The biosensor exhibited a LOD of 12 CFU·mL^−1^ but with a limited detection range of just two orders of magnitude. Although it is relatively narrow compared to the 5–7 orders of magnitude demonstrated for other GPEBs (Table 2), its ability to reliably detect trace contamination is more relevant for early risk assessment in foods than maximizing upper-range coverage.

The lowest LODs were achieved using DPV transduction [60]. In detecting *S. Typhimurium*, Ding’s biosensor [57] went below the 7 CFU·mL^−1^ achieved with qPCR [134]. While the high responsiveness of DPV likely played a role, other sensitive methods like EIS have not reached such low figures. As discussed above, Bhardwaj et al. reported a ~100-fold higher LOD, though this was likely due to a short incubation step. The other two reported impedimetric sensors achieved a LOD of 12 CFU·mL^−1^. Ding’s use of CRGO may have also contributed to improved detection by offering a higher surface density of carboxyl groups for RBP immobilization. These probes are more stable than whole phages, pose fewer orientation challenges, and reduce the area occupied by non-interacting structures like capsids or tail sheaths.

Nonetheless, CRGO is characterized by low conductivity and is often paired with conductivity-enhancing agents such as carbon black [58] or Au NPs [57]. The increased surface area and conductivity provided by Au NPs in Ding’s work is another possible key factor to its low LOD [57]. Yang et al. [60] also combined DPV transduction with performance-enhancing Au NPs, but used rGO instead of CRGO, which features higher electrical conductivity. Since the phages were physically immobilized, the higher density of carboxyl groups in CRGO would have brought no advantage. Selectivity as addressed in all of the reviewed studies, except for Quiton’s work [56]. While earlier studies evaluated this property with three to four non-host pathogens, more recent studies include a great variety of species or even serovars of the target bacterium.

Bhardwaj et al.’s *S. arlettae* sensor [51] was tested in the presence of non-host bacteria, including *S. aureus*, *S. lentus*, and Gram-negative *E. coli*. The *R_CT_*v alues registered upon incubation with the mixture of host and non-host species decreased by less than 0.3% compared to target-only incubation, confirming high selectivity to *S. arlettae*, even in the presence of other staphylococci. Keihan et al. claimed their biosensor showed selectivity against various Gram-negative and Gram-positive bacteria from the genera *Klebsiella*, *Shigella*, *V. cholerae*, and *S. aureus* [61], though no experimental data were provided. Nakama et al. [59] evaluated their biosensor with two controls: (1) the phage-functionalized GFET tested with *P. chlororaphis*, a non-host, which showed no change in resistance (gray circles, Figure 12); (2) a phage-free device tested against *E. coli* also supplied no response (empty circles).

**Figure 13:**
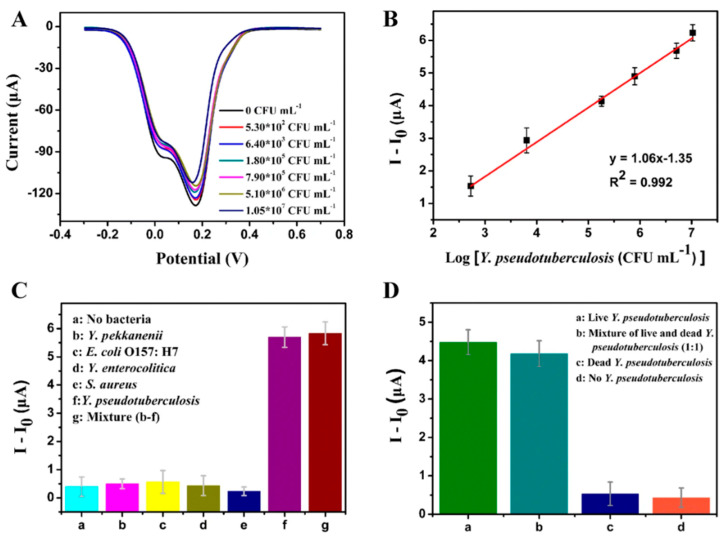
(**A**) Differential pulse voltammograms (DPVs) obtained in PBS (0.1 M, pH 6.0) for the GCE/PI-5-CA/rGO/AuNPs/phage electrode after incubation with solutions of *Y. pseudotuberculosis* with different cell density: 0–1.05 × 10^7^ CFU·mL^−1^. (**B**) Linear relationship found between the current inhibition factor (I-I_0_) and the logarithm of the concentration of *Y. pseudotuberculosis*. (**C**) Control experiments performed to evaluate the selectivity of the sensor towards other pathogens at the same concentration (3.00 × 10^6^ CFU·mL^−1^). (**D**) Capability to discriminate between live (3.30 × 10^5^ CFU·mL^−1^), dead (3.30 × 10^5^ CFU·mL^−1^), and a mixture of live/dead *Y. pseudotuberculosis* cells (1:1). Reprinted with permission from Q. Yang et al. [60]. Copyright 2021, Springer Nature.

These results confirmed that M13 phages can discriminate *E. coli* from non-host bacteria, validating the sensor’s specificity. The GCE/PI-5-CA/rGO/AuNPs/vB_YepM_ZN18 biosensor introduced by Yang et al. [60] was evaluated against *Y. pseudotuberculosis* and other *Yersinia* spp. (*Y. pekkanenii* and *Y. enterocolitica*), as well as bacteria from different genera (*S. aureus* and *E. coli*). As shown in Figure 13C, no significant change occurred in the response to non-target pathogens (Blocks b–e) compared to the non-incubated blank (Block a). When exposed to the target alongside non-hosts (Block g), the response remained comparable to that for the target alone (Block f). These findings highlight the remarkable specificity of vB_YepM_ZN18 phages for *Y. pseudotuberculosis*, enabling discrimination across genera and within *Yersinia*, down to the species level.

**Figure 14:**
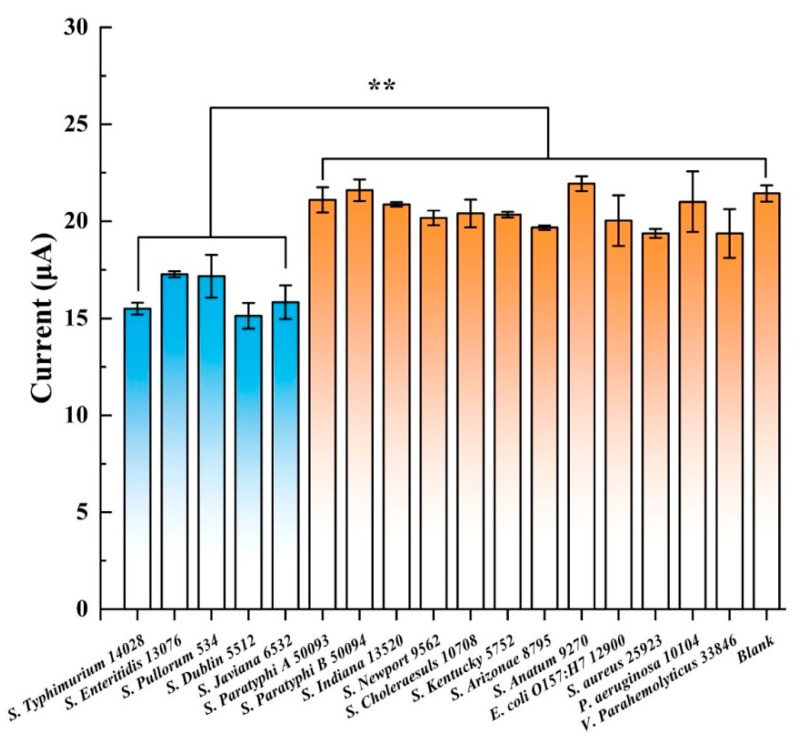
DPV currents obtained for a GCE/CRGO/AuNPs/RBP41 electrode incubated with different *Salmonella* spp. (including the target *S.* Typhimurium 14028) and other bacteria from different genera. All the pathogens were present at same concentration (10^5^ CFU·mL^−1^). ** denotes a statistically significant difference (*p* < 0.01) between the blue- and orange-coded pathogens. Reprinted with permission from Y. Ding et al. [57]. Copyright 2024 Elsevier Science Ltd.

Y. Ding et al. demonstrated the lytic capabilities of T102 phages against 23 different *Salmonella* strains [143]. Following this finding, they expressed and purified a key binding protein, RPB 41 (NCBI accession: UUG66264) [144], and constructed a *Salmonella* biosensor by covalently anchoring it onto CRGO [57]. The sensor was tested against *S.* Typhimurium 14028 (a previously identified T102 host) and twelve additional *Salmonella* spp. As shown in Figure 14, only four *S. enterica* strains—*S.* Enteritidis 13076, *S.* Pullorum 534, *S.* Dublin 5512, and *S.* Javiana 6532—produced current reductions comparable to *S.* Typhimurium 14028. Remarkably, all four were previously confirmed as T102 hosts [143]. Thus, while the sensor was not strictly specific to *S.* Typhimurium, it could selectively detect a narrow range of *Salmonella* strains. A sensor capable of simultaneously detecting multiple *Salmonella* serovars is particularly valuable as all these response-triggering strains—except *S.* Pullorum—pose significant risks to human health.

Also consistent with earlier observations [143], non-*Salmonella* pathogens tested negative (Figure 14), confirming T102 inactivity against them. These findings show how phage’s narrow host range can be leveraged in biosensors by using specific binding proteins. This strategy preserves the phage’s inherent specificity while improving sensor stability, durability, and analytical performance by enabling higher probe densities on the surface. The specificity tests conducted by Zhou et al. with their *E. coli* GXEC-N07 biosensor [58] were more limited, involving only bacteria from other genera, such as *K. pneumoniae*, *P. aeruginosa*, and *S. enteritidis*. Target detection caused a strong shift in the interfacial charge transfer resistance (∆*R_CT_*) of −2 kΩ, while interfering pathogens and the blank produced only minor changes (10–20 Ω), confirming the high affinity of the immobilized phages for *E. coli* GXEC-N07.

Unfortunately, the response to other strains or serovars, such as *E. coli* 614 or XL1-blue, was not evaluated, leaving it unclear whether the sensor is specific to GXEC-N07 or detects a broader *E. coli* range. Regarding the ability to differentiate live from dead pathogens—an essential feature previously discussed—this was only evaluated in the works of Q. Yang and W. Hussein [60,62]. Yang et al. prepared a 1:1 mixture of live and dead *Y. pseudotuberculosis* cells (3.30 × 10^5^ CFU·mL^−1^ each), with dead cells produced by autoclaving at 121 °C for 25 min [60]. The biosensor’s response to the mixture showed no significant difference from that with live cells alone (Figure 13D). Likewise, the response to only dead cells was comparable to the blank, confirming viability discrimination.

Hussein et al. tested their biosensor with heat-killed *S*. Typhimurium cells (100 °C for 25 min) and mixtures of live/dead cells, yielding minimal and intermediate responses, respectively [62]. Notably, it also achieved the lowest detection limit among all the reviewed sensors—1 CFU·mL^−1^—which, combined with its ability to detect only viable cells, underscores the effectiveness of the immobilized phages and the sensor’s transduction scheme. Shifting the focus to real-world applicability, most GPEBs have demonstrated practical performance in real or simulated food and water samples. Notable exceptions include devices developed by Quiton et al. and Keihan et al. [56,61], which do not assess these capabilities. In most cases, the standard addition method was used alongside colony-counting or ELISA to determine recovery rates.

Bhardwaj et al. reported recoveries ranging from 83% to 117% in river water samples and 93% to 126% in apple juice [51]. These figures broadly represent most sensor performances. Q. Yang et al. reported a narrower range (95–104%) [60], underscoring their platform’s reliability. The only study with recoveries below 80% was introduced by Y. Zhou et al. [58]. Figure 4 shows the results obtained from raw pork meat and milk, with the former yielding rates between 61 and 110%. Despite the robust design, these reduced values suggest that complex matrix effects [145]—likely interfering with phage–bacterium interactions at the sensing surface—remain a practical challenge for this sensor. Such effects, common in protein- and fat-rich samples [146,147], may reduce bacterial capture efficiency and explain the recovery loss observed in this study.

## 4. Conclusions and Future Perspectives

This review provides a comprehensive discussion of the existing literature on GPEBs, which predominantly target the quantitative detection of foodborne bacteria. These sensors combine the high specificity and straightforward production of phages with graphene’s exceptional electrical conductivity, biocompatibility, and large surface area. They have consistently detected bacterial loads as low as those reached by traditional microbiological methods and have frequently outperformed advanced techniques such as PCR, ELISA, and antibody- or aptamer-based EBs. Phage attachment—whether achieved through physical adsorption or covalent bonding—plays a key role in determining probe coverage and orientation on the sensor surface. However, current research does not support favoring one approach over another as both strategies have yielded comparable LODs and linear ranges. In addition, these comparisons are hampered by the limited physico–chemical characterization of the nanocomposites and, in particular, of the immobilized phages themselves. Crucial parameters, such as probe density, stability, or spatial orientation, are rarely reported, making it difficult to isolate the influence of immobilization strategy on biosensor performance at present time. The interplay between other factors—such as the electroactive surface area, nanocomposite conductivity, and the intrinsic responsiveness of the transduction method—further complicate this analysis.

GPEBs have demonstrated remarkable versatility, employing various electrochemical signals—voltametric, impedimetric, capacitive, and resistive—to enable on-site pathogen quantification. Among these, the most sensitive biosensors to date have utilized differential pulse voltammetry, achieving detection limits below 5 CFU·mL^−1^. Additionally, GPEBs offer significantly faster response times—ranging from seconds to 35 min—than conventional laboratory methods, while covering pathogen cell densities across multiple orders of magnitude. Incorporating graphene consistently enhances electrode performance, while specific phages (or their binding proteins) enable selective recognition of target bacteria—even in complex samples containing different strains from the same genus.

**Figure 15:**
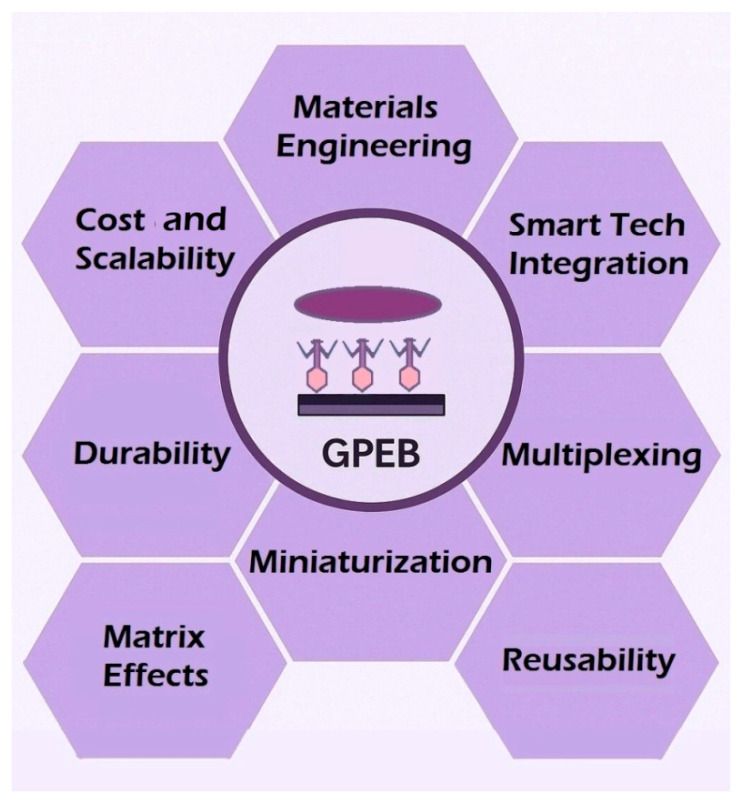
Overview of the main bottlenecks limiting the broader implementation of GPEBs. These include challenges related to material selection, probe immobilization, sensor reusability, integration with digital systems, and operation in real-world environments. Each topic is addressed in detail in the concluding section.

Furthermore, GPEBs’ ability to distinguish live from dead cells, as demonstrated in two studies, is crucial for accurate contamination risk assessment. Although research into graphages is still emerging, their impressive analytical performance and cost-effectiveness suggest substantial growth in GPEB research in the coming years. However, many of the factors that will ultimately define the robustness and practical usability of these sensors remain underexplored. Figure 15 illustrates the major challenges and research priorities emerging from the current state of GPEB development. These encompass critical aspects ranging from the exploration of novel materials to system integration and practical deployment. The most relevant of these bottlenecks are addressed in the following points:

(a) Materials Engineering Aspects: The combination of phages with unexplored graphene derivatives—such as heteroatom-doped or graphene–nanoparticle conjugates—and the use of conductivity-enhancing agents (e.g., metal or semiconductor NPs, ionic liquids, or conducting polymers) could further amplify detection capabilities. Improvements in surface modification techniques could lead to enhanced probe immobilization efficiency and optimized phage orientation for better pathogen recognition. These innovations could potentially push GPEBs’ detection limits below 1 CFU·mL^−1^. While phage display has not yet been applied in reported GPEBs, it holds considerable potential for future integration [148]. By enabling genetically engineered phages with site-specific attachment tags, phage display could enhance immobilization stability, facilitate orientation control, and support modular receptor design for improved selectivity. These advantages may also contribute to improved reproducibility and cost-efficiency in complex sample conditions. For a more detailed overview of synthetic biology tools in phage-based biosensors, readers are referred to recent specialized reviews such as that reported by Wang et al. [91].
(b) Cost and Scalability: Traditional detection methods, such as PCR and ELISA, face significant economic and scalability challenges. PCR, while highly sensitive, requires sophisticated laboratory infrastructure, expensive reagents, and trained personnel, making it impractical for widespread, on-site applications. Similarly, ELISA relies on monoclonal antibodies, which are not only expensive, but are also difficult to produce at scale, further limiting its accessibility. Electrochemical immunosensors and aptasensors offer improved detection limits and portability but also face cost-related drawbacks. Immunosensors rely on monoclonal antibodies, while custom aptamers, although highly specific and versatile, remain prohibitively expensive to synthesize [21,22], limiting their commercial adoption. In comparison, bacteriophages represent a simpler and less expensive alternative as recognition probes. Nonetheless, the labor-intensive and costly process of large-scale phage purification remains a significant bottleneck for commercialization. Improving the efficiency of this step is essential to enable scalable production in the medium term. Employing RBPs, which can be more easily produced through recombinant overexpression [149], also offers a promising solution to overcome this challenge. Additionally, developing strategies for recovering and reusing immobilized phages or RBPs in disposable devices could further reduce material costs and improve economic feasibility.
(c) Sensor Stability and Durability: Long-term stability of immobilized phages under varying environmental conditions remains largely underexplored. Bacterial lysis following recognition and capture can destabilize the sensor signal, particularly in impedance-based platforms [150]. The rupture of large bacterial cells exposes substantial portions of the sensor surface, causing unpredictable signal fluctuations. Exploring phage’s binding proteins as alternative probes to whole phages could address lysis issues while enhancing overall sensor durability in practical applications (thanks to the greater physicochemical stability of RBPs under extreme conditions).
(d) Expanding Multiplexing Capabilities: Early detection of multiple serovars in a single test could help contain outbreaks more rapidly by minimizing diagnostic delays. Except for Y. Ding et al.’s sensor [57], which responded to four *S. enterica* strains, current GPEBs typically focus on single-pathogen detection, limiting their practicality for broader applications. Future designs should enable simultaneous detection of various species or strains in a single test, significantly reducing time and resource requirements. Multiplexing could be achieved by co-immobilizing phages or RBPs targeting different genera or serovars. The smaller size of RBPs compared to whole phages may facilitate the assembly of a higher density and diversity of probes, further supporting multiplexing efforts. Co-immobilization with complementary recognition probes, such as antibodies or aptamers, is another possibility. Alternatively, multiple single-target GPEBs could be integrated into multichannel platforms.
(e) Miniaturization and Integration: While miniaturized GPEB designs using SPEs have been demonstrated, further standardization is crucial to advance portable, cost-effective devices for real-time, on-site pathogen monitoring. Miniaturization not only facilitates on-site practical application, but also reduces maintenance requirements and enhances sensor durability, particularly in harsh environments.
(f) Smart Integration: Linking GPEBs to cloud networks and integrating AI for real-time data analysis could enable continuous pathogen monitoring in food production, water systems, or healthcare applications. Integrated systems could provide predictive insights for early intervention during outbreaks but require substantial investments in infrastructure and standardization to ensure compatibility and reliability.
(g) Matrix Effects in Real Samples: Matrix effects remain a significant challenge when applying GPEBs to complex food and environmental samples. High protein or fat content, dense tissue matrices, and interfering compounds can hinder pathogen accessibility to immobilized phages or RBPs, reducing detection capabilities and analytical accuracy [146,147]. Addressing these effects will likely require the implementation of sample pre-treatment strategies, involving processes such as dilution, filtration, emulsion, selective enrichment, or matrix cleanup, to reduce the presence of interfering species without compromising target detection [151]. However, such approaches must be carefully optimized, as sample processing could affect the concentration of bacterial analytes. Signal modulation approaches may help correct for background noise and variability in complex matrices. In addition to internal standards or matrix-specific calibration models, software-based techniques such as chemometric data processing, machine learning algorithms, and wavelet-based signal filtering have shown promise in enhancing signal-to-noise ratios without requiring hardware modification [152,153]. Although antifouling coatings (such as sol-gel and PEG-based or zwitterionic layers) could minimize nonspecific adsorption [154], they may also hinder bacterial access to the sensing surface or interfere with phage orientation, potentially introducing further disadvantages in whole-cell biosensing applications.

In conclusion, GPEBs represent a promising platform for next-generation pathogen detection. With ongoing innovations in nanotechnology and electrochemical platform miniaturization, significant improvements in detection performance, specificity, multiplexing, and integration with smart technologies are expected. Although phages are simpler to produce than other probes, purification remains costly and labor-intensive. Hence, overcoming these manufacturing hurdles will be one key to enabling broader adoption. Using receptor binding proteins instead of phages could mitigate some of these challenges, but further research is required to develop scalable and cost-effective production methods. Additionally, developing strategies for recovering and reusing phages in disposable devices could further reduce economic barriers and enhance sustainability.

## Figures and Tables

**Table 1:** Overview of the composition, structure, target species, and practical applicability of the main graphage-based electrochemical and electrical biosensors documented in the literature to date.

GNM	Phage	Immobilization	Incubation Times	Biosensor Structure—Electrode	Real Samples	Transduction	Ref.
Electrochemically Oxidized Pristine Graphene (EOPG)	Anti-*Staphylococcus arlettae*	Covalent	2 h	EOPG/phage—SPE	River Water and Apple Juice	Impedance	[51]
GO	Anti-*Salmonella* Typhimurium	Covalent	6 h	GO/phage—SPE	None	Impedance	[56]
Carboxyl Rich GO (CRGO)	RBP 41 from Anti-*Salmonella* Typhimurium T102	Covalent	2 h	CRGO/AuNPs/phage—RBP—GCE	Milk and Lettuce	Voltammetry	[57]
CRGO	Anti-*Escherichia coli* EP01	Covalent	Overnight	CRGO/CB/phage—GCE	Milk and Raw Pork	Impedance	[58]
rGO	Anti-*E. coli* M13	Covalent	3 h	rGO/phage—FET	Simulated River Water	Resistance	[59]
rGO	Anti-*Yersinia pseudotuberculosis* vB_YepM_ZN18	Physical: Adsorption	2 h	PI-5-CA/rGO/AuNPs/phage—GCE	River Water	Voltammetry	[60]
rGO	Anti-*E. coli*	Physical: Adsorption	Does not apply	rGO/phage—CPE	No	Capacitance	[61]
rGO	Anti-*Salmonella* Typhimurium	Physical: Adsorption	Overnight	BC/PPy/rGO/phage–SPE	Milk and Chicken	Voltammetry	[62]

**Table 2:** Analytical performance of the genosensors, PEBs, PCR/ELISA methods, flow cytometry, culturing methods, and GPEBs, in the detection of bacterial pathogens.

Ref.	Target	Linear Range (CFU·mL^−1^)	Detection Method	Response Time	Verified Non-Interferent Species	LOD (CFU·mL^−1^)	Discerns Live/Dead Cells
[16]	*Salmonella* Typhimurium *	1.0 × 10–1.0 × 10^8^	DPV	~5 min	*5 S. entérica* serovars 5 non-*Salmonella* bacteria	10	No
[17]	*P. aeruginosa* ATCC 27853	60.0–6.0 × 10^7^	Amperometry	<10 min	S. aureus * V. cholerae *	60	No
[33]	*E. coli* K12	1.0 × 10^2^–1.0 × 10^8^	EIS	<30 min	*S.* Typhimurium DT108	10^4^	Non-tested
[34]	*L. monocytogenes* Scott A	10.0–1.0 × 10^4^	EIS	<30 min	*S.* Typhimurium 291RH *E. coli* O157:H7	8	Non-tested
[51]	*S. arlettae*	2.0 × 10^2^–2.0 × 10^8^	EIS	2 min	*S. aureus* 96 *S. lentus* 2292 *E. coli* 614	200	Non-tested
[56]	*Salmonella* Typhimurium	1.0 × 10–1.0 × 10^8^	EIS	<40 min	None	12	Non-tested
[57]	*Salmonella* Typhimurium ATCC14028	3.0–1.0 × 10^6^	DPV	~30 min	8 *Salmonella* spp. 4 non-*Salmonella* bacteria	2	Non-tested
[58]	*E. coli* O157:H7GXEC-N07	1.0 × 10^2^–1.0 × 10^7^	EIS	<30 min	*P. aeruginosa* PAI *Klebsiella pneumoniae* L30 *S. enteritidis* CVCC1806	12	Non-tested
[59]	*E. coli* XL1-blue	1.0 × 10^2^–1.0 × 10^7^	FET	30 min	*P. Chlororaphis*	45	Non-tested
[60]	*Y. pseudotuberculosis*	5.3 × 10^2^–1.1 × 10^7^	DPV	35 min	Y. enterocolítica * Y. pekkanenii * S. aureus * E. coli O157:H7	3	Yes
[61]	E. coli *	3.3 × 10–3.3 × 10^2^	LCR	5 s	S. aureus * Klebsiella * Shigella * V. cholerae *	12	Non-tested
[62]	*Salmonella* Typhimurium	1.0–1.0 × 10^7^	DPV	30 min	S. aureus* L. monocytogenes * E. coli * Bacillus subtilis * S. Typhimurium (heat-killed)	1	Yes
[133]	*E. coli* ATCC 25922	1.0 × 10^3^–1.0 × 10^5^	FET	50 s	Heat-killed: *S.* Typhimurium *Streptococcus pneumonia*	10^3^	Non-tested
[134]	*Salmonella* Typhimurium ATCC14028	7.0–7.0 × 10^5^	qPCR	<3 h	4 *S.* Typhimurium serovars 3 other *Salmonella* spp. 5 non-*Salmonella* bacteria	7	No
[135]	*Salmonella* Typhimurium DB7155 and 20 other *Salmonella* spp.	1.0 × 10^5^–1.0 × 10^7^	ELISA	2 h	10 non-*Salmonella* bacteria	100	No
[136]	*E. coli* and other 9 bacteria	-	Flow Cytometry	21–64 h	-	10^5^	No
[137]	*Salmonella* spp.	-	Bacterial Culture (ISO 6579)	58–74 h	*-*	1	Yes

* serovars or serotypes not specified.

## Data Availability

No new data were created or analyzed in this study.

## Abbreviations

Au	Gold
AFM	Atomic Force Microscopy
APTES	(3-Aminopropyl)triethoxysilane
APTMS	(3-Aminopropyl)trimethoxysilane
BC	Bacterial Cellulose
BPs	Bacterial Pathogens
BSA	Bovine Serum Albumin
CB	Carbon Black
CFU	Colony-Forming Units
CPE	Carbon Paste Electrode
CRGO	Carboxyl-Rich Graphene Oxide
CV	Cyclic Voltammetry
DPV	Differential Pulse Voltammetry
EBs	Electrochemical Biosensors
EDC	1-Ethyl-3-(3-dimethylaminopropyl) Carbodiimide
EFSA	European Food Safety Agency
EIS	Electrochemical Impedance Spectroscopy
ELISA	Enzyme-Linked Immunosorbent Assay
EOPG	Electro-Oxidized Pristine Graphene
FC	Flow Cytometry
FET	Field-Effect Transistor
GCE	Glassy Carbon Electrode
GEBs	Graphene-Based Electrochemical Biosensors
GFET	Graphene-Based Field-Effect Transistor
GNMs	Graphene Nanomaterials
GO	Graphene Oxide
GPEBs	Graphage-Based Electrochemical Biosensors
LOD	Limit of Detection
LCR	Low-Frequency Capacitance/Inductance
MES	2-(N-Morpholino)ethanesulfonic Acid (MES buffer)
NCBI	National Center for Biotechnology Information
NHS	N-Hydroxysuccinimide
NPs	Nanoparticles
PCR	Polymerase Chain Reaction
PD	Phage Display
PEBs	Phage-Based Electrochemical Biosensors
PEI	Polyethylenimine
PI-5-CA	Polyindole-5-carboxylic acid
PLL	Poly-L-Lysine
PPy	Polypyrrole
PVA	Polyvinyl Alcohol
PDADMAC	Poly(Diallyldimethylammonium Chloride)
qPCR	Quantitative Polymerase Chain Reaction
RBPs	Receptor Binding Proteins
rGO	Reduced Graphene Oxide
SAMs	Self-Assembled Monolayers
SEM	Scanning Electron Microscopy
SPEs	Screen-Printed Electrodes
